# Thrombotic, Cardiovascular, and Microvascular Complications of Myeloproliferative Neoplasms and Clonal Hematopoiesis (CHIP): A Narrative Review

**DOI:** 10.3390/jcm13206084

**Published:** 2024-10-12

**Authors:** Andrew I. Schafer, Douglas L. Mann

**Affiliations:** 1 Richard T. Silver MPN Center, Department of Medicine, Weill Cornell Medical College, New York-Presbyterian Hospital/Weill Cornell, New York, NY 10021, USA; 2 The Center for Cardiovascular Research, Washington University, St. Louis, MO 63110, USA; dmann@wustl.edu

**Keywords:** myeloproliferative neoplasms, clonal hematopoiesis of indeterminate potential, thrombosis, microvascular

## Abstract

The most common causes of morbidity and mortality in the myeloproliferative neoplasms (MPNs), with the exception of myelofibrosis, are venous and arterial thrombosis, as well as more recently discovered cardiovascular disease (CVD). Clonal hematopoiesis of indeterminate potential (CHIP) is the subclinical finding in an individual of somatic mutations that are also found in clinically overt MPNs and other myeloid malignancies. The prevalence of “silent” CHIP increases with age. CHIP can transform into a clinically overt MPN at an estimated rate of 0.5 to 1% per year. It is likely, therefore, but not proven, that many, if not all, MPN patients had antecedent CHIP, possibly for many years. Moreover, both individuals with asymptomatic CHIP, as well as clinically diagnosed patients with MPN, can develop thrombotic complications. An unexpected and remarkable discovery during the last few years is that even CHIP (as well as MPNs) are significant, independent risk factors for CVD. This review discusses up-to-date information on the types of thrombotic and cardiovascular complications that are found in CHIP and MPN patients. A systemic inflammatory state (that is often subclinical) is most likely to be a major mediator of adverse reciprocal bone marrow–cardiovascular interplay that may fuel the development of progression of MPNs, including its thrombotic and vascular complications, as well as the worsening of cardiovascular disease, possibly in a “vicious cycle”. Translating this to clinical practice for hematologists and oncologists who treat MPN patients, attention should now be paid to ensuring that cardiovascular risk factors are controlled and minimized, either by the patient’s cardiologist or primary care physician or by the hematologist/oncologist herself or himself. This review is intended to cover the clinical aspects of thrombosis and cardiovascular complications in the MPN, accompanied by pathobiological comments.

## 1. Introduction

The classical Philadelphia chromosome-negative myeloproliferative neoplasms (MPNs) encompass a group of related hematopoietic stem cell disorders that include essential thrombocythemia (ET), polycythemia vera (PV), and primary myelofibrosis (PMF). The natural history of these disorders often varies from *overlap* syndromes (e.g., pre-fibrotic myelofibrosis, MPN/myelodysplasia overlap), *transitions* (e.g., ET to PV), or *disease progression* (e.g., post-ET myelofibrosis, post-PV myelofibrosis, or acute leukemia). While hematopoietic stem cell transplantation is currently the only potential cure, the armamentarium of drugs to control MPNs is undergoing remarkable expansion.

In most cases of MPNs, so-called “driver mutations” develop, apparently spontaneously, to trigger the clinical disease. These variants include *JAK2 V617F*, *JAK2 exon 12/13*, *calreticulin* (*CALR*), and *MPL W51F.* Although these variants are somatic mutations that are not inherited in the great majority of MPN patients, they can appear as early in life as at 33 weeks of gestation and lie dormant for decades, if not for a lifetime [1]. These variants, when they are clinically overt “drivers”, can largely dictate clinical phenotypes, such as thrombocytosis, polycythemia (erythrocytosis), leukocytosis, or pancytosis. In a few cases of isolated thrombocytosis, chronic myelogenous leukemia (CML) can masquerade as ET, requiring an entirely different treatment approach. Therefore, when genomic screening for MPN is carried out for a patient with unexplained, isolated thrombocytosis, testing for *BCR-ABL1* (associated with CML) should not be neglected. About 10% of patients with ET or PMF do not harbor any of these driver mutations; they are referred to as “triple negative” MPNs [2]. In these cases, bone marrow aspirate and biopsy become even more crucial than otherwise to make a definitive diagnosis.

The major causes of morbidity and mortality in the MPNs are thrombotic and cardiovascular or microvascular complications. Morbidity and mortality from these MPN complications well exceed morbidity/mortality rates caused by MPN progression of disease. For example, in one study, the cause of mortality in PV patients from thrombotic (and bleeding) events was 45%, while it was only 13% from MPN disease progression to myelofibrosis and/or acute leukemia [3].

## 2. Clonal Hematopoiesis (CHIP)

Hematopoietic stem cells accumulate random mutations as a function of normal aging. The majority of these mutations are so-called “passenger mutations” that have no known pathogenic significance, in contrast to the “driver mutations” found in CHIP and MPNs (as well as other hematologic malignancies). The concept of clonal hematopoiesis of indeterminate potential (CHIP) emerged over a decade ago. CHIP is strongly linked to the MPNs. CHIP is defined as follows: (1) the presence in peripheral blood of hematopoietic cells with somatic mutations known to be responsible for hematologic malignancies (including the MPNs), such as *JAK2 V7617F*, *TET2*, *ASXL1*, *EZH2*, *IDH1*, and *DNMT3A*; (2) having variant allele frequencies (VAF) of 2% or higher; (3) the *absence* of clinically overt hematologic malignancies (including MPNs); and (4) the *absence* of criteria for other preleukemic states [4]. CHIP is now recognized as a disorder of aging. By the age of 70, about 10–20% of otherwise healthy individuals harbor a peripheral blood cell mutant clone with a VAF of ≥2% [4]. By age > 85, the prevalence is ≥50% [5].

Individuals with CHIP in peripheral blood cells have been considered to have an approximately 10-fold increased risk of developing clinically overt MPN or other hematologic malignancies [5,6]. Clonal *cytopenia* of undetermined significance (CCUS) is another form of clonal hematopoiesis (CH) [7]. The overall rate of transformation of CH (CHIP *and* CCUS) to MPN or other hematologic malignancy, is approximately 0.5 to 1% per year [8]. To distinguish patients who are at especially high risk of progression from CHIP to clinically overt MPN, prognostic platforms and models have been proposed. Their aim is to distinguish individuals with CHIP who are at a particularly high-risk *minority* from the large *majority* of individuals with CHIP who have only a minimal risk of progression to clinically overt MPNs [8].

## 3. CHIP Associated with Cardiovascular Disease

While the initial findings were that CHIP and MPNs promote atherogenesis and, conversely, the inflammatory state that accompanies atherosclerosis feeds back to the bone marrow to selectively promote mutant hematopoietic stem cell proliferation, more recent studies have found that other forms of cardiovascular disease, particularly heart failure, promote the same adverse feedback loop, as described below [6]. CHIP found in peripheral blood cells, detected by next-generation DNA sequencing, originates in the bone marrow. However, with the expansion of a mutant clone in the marrow (accelerated by its preferential proliferation over normal stem cells), mutant stem cells release circulating, mature progeny, including red cells, platelets, leukocytes, and other immune cells that also harbor the same mutation [9]. The proliferation of mutant hematopoietic stem cells is accelerated by their progressive dominance over normal stem cells.

The most exciting and unexpected offshoot of the finding of CHIP in older individuals has been the discovery of a striking (over two-fold) cardiovascular risk in individuals who harbor a CHIP (compared to those who do not). CHIP is now known to be an independent cardiovascular risk factor, comparable in magnitude to established cardiovascular risks like hypercholesterolemia, diabetes mellitus, hypertension, smoking, and obesity. CHIP was at first associated with only coronary artery disease in humans and with accelerated atherosclerosis in mice [10]. Subsequently, it was found that CHIP predisposes to other cardiovascular disorders, including ischemic stroke [11], degenerative aortic valve stenosis, peripheral artery disease [12], and, most notably, heart failure (see below). Clonal hematopoiesis has also been found to contribute to all-cause mortality [13].

Epidemiologic studies have shown a robust link between clonal hematopoiesis and heart failure, independent of traditional risk factors [14]. In a meta-analysis of >56,000 subjects who were free of heart failure at baseline and carriers of a CHIP mutation had a 25% increased risk of incident heart failure. Individuals who are carriers of CHIP (especially *TET2*-driven) were found to have worsened diastolic heart function, along with a poorer prognosis in heart failure with reduced ejection (HFrEF) [15,16]. Somatic mutations that drive clonal hematopoiesis are common (approximately 50%) among heart failure patients with reduced left ventricular ejection fraction and are associated with accelerated heart failure progression and worse clinical outcomes [17]. Recent studies have also suggested a pathogenic role of CHIP in heart failure patients with a preserved ejection fraction (HFpEF), who have worse diastolic dysfunction and a higher rate of cardiovascular-related hospitalizations when compared to HFpEF patients without CHIP [17].

The pathophysiological mechanisms that link CHIP to HFrEF involve chronic myocardial inflammation that is caused by the influx of proinflammatory immune cells into the myocardium. In CHIP-associated HFrEF, a wide range of immune cells contribute to systolic dysfunction and adverse remodeling. In contrast, HFpEF is characterized by a more targeted infiltration of proinflammatory macrophages and T cells, resulting in diastolic dysfunction and fibrosis [17,18].

## 4. Myeloproliferative Neoplasms Associated with Cardiovascular Disease

If CHIP alone confers an increased risk of accelerated progression of atherosclerosis and heart failure, other cardiac disorders, and related arterial thrombosis, then surely the clinically diagnosed MPNs should be associated with at least the same risk. Indeed, there is an increased overall cardiovascular risk in patients with clinical MPNs [19]. Up to 75% of patients with MPNs experience a major adverse cardiovascular event, presumably as a complication of their MPNs. In addition, about a third of MPN patients who have had an acute coronary syndrome will have another major cardiovascular event [20].

Myeloproliferative neoplasms and heart failure: Patients with MPNs have an increased risk of heart failure or heart failure progression, particularly high-output heart failure (HOHF).^B^ A large Medicaid database study showed a >2-fold increased risk for heart failure in patients with MPNs compared with those without MPNs [21,22]. The mechanisms for high-output states are thought to be increased oxygen demand secondary to splenomegaly and hepatomegaly that are caused by extramedullary hematopoiesis, as well as a reduction of systemic vascular resistance caused by increased circulatory proinflammatory and angiogenic cytokines, such as interleukin (IL)-6, IL-8, tissue necrosis factor-alpha, and vascular endothelial growth factor. Proinflammatory cytokines can also lead to myocardial inflammation and fibrosis, exacerbating underlying heart failure.

*Myeloproliferative Neoplasms and Pulmonary Hypertension*

Two recent studies estimated a 4% to 7% prevalence of pulmonary hypertension in patients with MPNs [23,24]. Overall, the prevalence of pulmonary hypertension in the MPNs has been estimated to range widely from 4 to 58% [25,26]. Inflammation has been linked to pulmonary arterial stiffening [27].

## 5. Inflammation as the Key Mediator of the Bone Marrow–Cardiovascular Axis

**Figure 1:**
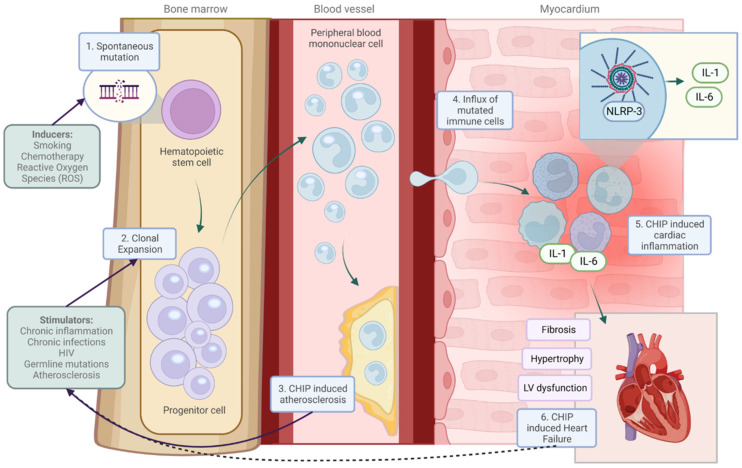
The association between clonal hematopoiesis and heart failure. Mutations in hematopoietic stem cell progenitor cells give rise to clones that expand over time. (1) Factors stimulate clonal proliferation; (2) consequently, these mutated cells enter the bloodstream and myocardium and cause atherosclerosis, (3) or impair cardiac function. (4) An inflammasome/interleukin 1/6-mediated response (5) is central to clonal hematopoiesis-induced heart failure. (6) Heart failure could be a driver of clonal proliferation, as indicated by the dashed line. Solid lines are based on published results. CHIP indicates “clonal hematopoiesis of indeterminate potential”. IL-1, interleukin-6; LV, IL-6, interleukin-6; LV, left ventricular; NLRP3, NLR family pyrin domain-containing 3; ROS, reactive oxygen species [30].

Individuals with a CHIP or MPNs, particularly those with JAK2 mutations, produce excessive cytokines and reactive oxygen species that together lead to a progressively pro-inflammatory milieu in the bone marrow microenvironment of hematopoietic stem cells [20], which will then enter the systemic circulation. Conversely, pro-inflammatory cytokine levels are found to be increased in heart failure [28] and also in atherosclerosis [28,29] (e.g., from atherosclerotic macrophages), irrespective of CHIP or MPN. This then creates and sustains a systemic pro-inflammatory state throughout the body. Conceptually, it is likely that there is a reciprocal cycle of adverse pro-inflammatory factors between the bone marrow and the cardiovascular system: inflammatory cytokines are produced by the bone marrow microenvironment of MPN mutant hematopoietic stem cells that enter the circulation and adversely affect heart failure and atherosclerosis. At the same time, those cardiovascular diseases themselves produce inflammatory signals, which fuel the selective proliferation of mutant hematopoietic stem cells, out-competing normal stem cell growth. A vicious cycle of inflammation then ensues bidirectionally between the bone marrow and the cardiovascular system (Figure 1).

## 6. Arterial Thrombosis in the Myeloproliferative Neoplasms

In general, most individuals who have arterial thrombosis (with or without MPN) have underlying atherosclerotic cardiovascular disease, referred to as “atherothrombosis”. The sites of arterial thrombosis in these patients are typically where there is atherosclerotic plaque accumulation and rupture that exposes highly thrombogenic substances on the intimal surfaces of the arteries, causing localized activation of circulating platelets and coagulation proteins. Nonatheromatous arterial thrombosis is relatively rare [31]. Venous thromboembolism, in contrast, does not involve atherosclerosis.

A major study conducted from comprehensive population health data in Sweden between 1987 and 2009, with follow-up in 2010, assessed thrombosis risk among approximately 9500 MPN patients (with PV, ET, PMF, or MPN unclassified) and 35,000 matched controls. The hazard ratios of specifically *arterial* thrombosis among MPN patients compared to controls at 3 months and 1 and 5 years after MPN diagnosis were 3.0, 2.0, and 1.5, respectively. The incidence of arterial and venous thromboembolism was strikingly highest at the time of MPN diagnosis or shortly after it [32]. The decline in the incidence of thrombosis over time after MPN diagnosis has been attributed to good control of the disease and appropriate antithrombotic prophylaxis. There is clearly an increased risk of cardiovascular disease in patients with MPNs [18]. The rate of ischemic cerebrovascular events in MPN patients is approximately 10-fold higher than in the general population [33].

Peripheral arterial disease (PAD) is also not uncommonly seen in patients with MPN, especially those who are elderly. In a study of over 1000 MPN patients, 7.3% developed PAD [34]. In addition to aging, increasing the risk for PAD has been noted with diabetes mellitus, dyslipidemia, and tobacco smoking; all of these traditional risks are treatable. Clinical symptoms are typically leg pain, cramps, and intermittent claudication. Untreated PAD can result in gangrene of toes and feet, often requiring amputation (see section on digital ischemia).

Although cardiovascular physicians, vascular surgeons, and neurologists have traditionally been the specialists who primarily deal with cardiovascular, cerebrovascular, and peripheral artery occlusion risk factors in patients in general, the growing recognition of the need to optimize such risks in patients with MPN (or even CHIP) has made their recognition and management an integral part of a hematologist’s or oncologist’s overall management of patients with MPNs, ideally in close collaboration with the cardiovascular and stroke specialists. In remote areas where such specialists are very difficult to access, the hematologist/oncologist and/or the primary care physician should assume even greater responsibility for managing these risk factors that are now recognized to be so critical in providing excellent care for MPN patients.

Philadelphia chromosome-negative MPN patients and even individuals with CHIP are prone to the development of second cancers [35]. In a case–control study, it was found that, after adjustment for confounders, the occurrence of arterial thrombosis remained independently associated with the risk of second malignancy. Therefore, it is recommended that MPN patients who have had arterial events following MPN diagnosis should have surveillance for early detection of cancers [36].

## 7. Venous Thromboembolism in the Myeloproliferative Neoplasms

The most common form of venous thromboembolism (VTE) in the MPNs is “garden variety” deep vein thrombosis (DVT) of the lower extremities and/or pulmonary embolism (PE), clinically indistinguishable from VTE in patients without MPN. Treatment and then prophylactic antithrombotic therapy in this group of patients with MPNs specifically has been only incompletely addressed in evidence-based guidelines. As for the choice of anticoagulant, low-molecular heparin and warfarin were favored in the past, but this has been more recently eclipsed by the wide use of direct oral anticoagulants (DOACs) [37,38]. Regardless of the anticoagulant used for thrombosis, it should be considered that patients with MPN can also have an intrinsic bleeding tendency that could be worsened by anticoagulation. In MPN patients with very high platelet counts, the *acquired* von Willebrand syndrome with bleeding (like the inherited form) can develop, which tends to resolve when the platelet count is sufficiently lowered.

With optimally controlled anticoagulation, specifically using a DOAC, there is no complete assurance that a recurrence of thrombosis will not occur. In an observational, multicenter, international study of 442 patients with MPN, VTE occurred in 9.2% of patients while on a DOAC. There was also clinical bleeding after starting DOAC in 5.0% of patients [39].

Unfortunately, there have been no rigorous clinical studies that dictate the *duration* of secondary prophylaxis required for MPN patients for recurrent thrombosis. Many MPN clinicians follow the solid tumor cancer guidelines in this respect, accepting that MPNs are also neoplasms. For solid tumor cancers, the duration of anticoagulation after thrombosis, as recommended by the American Society of Clinical Oncology (ASCO) is usually 3–6 months, but can be longer. The American Society of Hematology (ASH) suggests long-term anticoagulation for secondary prophylaxis. Consensus guidelines generally recommend continuing anticoagulation in patients with active cancer or receiving cancer treatment until there is no evidence of disease, with periodic reassessment of the risk of recurrent thrombosis versus bleeding balance.

It is difficult to simply extrapolate to MPNs from the guidelines for antithrombotic prophylaxis in patients with solid tumor cancer-associated VTE. First, the effects of active MPN are lifelong unless a successful bone marrow stem cell transplant is achieved. Therefore, it follows that prophylactic anticoagulation for MPN patients should be strictly speaking lifelong. Second, unlike most solid tumor cancers, the MPNs are intrinsically associated with bleeding risk. Therefore, it would be prudent to anticoagulate a patient with MPN with prior thrombosis on an individual basis, repeatedly assessing the risk or rethrombosis without anticoagulation, as opposed to the risk of bleeding with continued anticoagulation (e.g., with falls in the elderly) [40,41,42,43].

For initial treatment of VTE in MPN patients, the evidence-based guidelines are essentially the same as for patients without MPN, who have acute VTE [44,45]. The caveat in MPN patients is that the disease itself carries an inherent risk for bleeding, not only thrombosis.

## 8. Unusual Sites of Thrombosis in the Myeloproliferative Neoplasms

While deep vein thrombosis with or without pulmonary embolism is by far the most common form of venous thrombosis in these patients, some other, more unusual sites of thrombosis are characteristics of the MPNs, although they are by no means diagnostic. Some of those unusual sites are splanchnic vein thrombosis, cerebral venous sinus thrombosis, and retinal vein obstruction [46].

**Splanchnic vein thrombosis.** The splanchnic venous circulation encompasses the hepatic and portal veins, with extension into the mesenteric and splenic veins. Hepatic vein thrombosis is also referred to as the Budd–Chiari syndrome. The most common causes of splanchnic vein thrombosis (SVT) in the general population include hepatic cirrhosis, post-liver transplant, and other liver diseases, blunt force trauma, local intra-abdominal processes such as malignant or benign tumors of the hepatobiliary system, abdominal surgery or abscesses, contiguous inflammatory disorders such as cholecystitis, pancreatitis, peritonitis, inflammatory bowel disease (especially when active), diverticulitis, and abscesses. Systemic disorders that can cause splanchnic vein thrombosis include thrombophilia, paroxysmal nocturnal hemoglobinuria (PNH), and pregnancy or other hormonal changes (e.g., oral contraceptives, hormone-replacement therapy).

In primary splanchnic vein thrombosis, where none of the above-listed causes are identified, the most common etiology is MPNs; MPNs comprise almost 40% of patients with Budd–Chiari syndrome and portal vein thrombosis. Splanchnic vein thrombosis is also a frequent presenting manifestation of MPN. MPNs associated with splanchnic vein thrombosis show a predilection for younger women and the *JAK2 V617F* mutation with a low variant allele frequency (generally < 10%) [47]. Asymptomatic splanchnic vein thrombosis is not uncommon. The diagnosis in these patients is usually made by their coincidental findings in imaging studies that were performed for other reasons. With subacute or chronic splanchnic vein thrombosis portal hypertension develops, leading to congestive splenomegaly and hypersplenism. Pancytopenia typically occurs with hypersplenism. Therefore, in these cases, the tip-off that an underlying MPN is present is the finding of inappropriately *normal* blood counts [48,49,50,51].

The management of MPN patients who have splanchnic vein thrombosis should involve an interdisciplinary team of hematologists with expertise in MPNs, hepatologists, interventional radiologists, and liver transplant specialists. Most of the treatments in these patients are comparable to the treatment of splanchnic vein thrombosis in patients without MPNs. The aims of prompt treatment are to prevent thrombus progression, promote vessel recanalization, and prevent recurrent thrombosis [51]. Early anticoagulation is mandatory. One study reported that partial or complete recanalization occurred in 71% of patients who started anticoagulation within 2 weeks from diagnosis of splanchnic vein thrombosis, versus 40% of those who started anticoagulation later [52]. Unfortunately, there are no evidence-based guidelines regarding the choice of anticoagulant and duration of anticoagulation; and there are even fewer data guiding treatment of splanchnic vein thrombosis in MPN patients. In general, recommendations have been to administer promptly started, long-term, indefinite-duration anticoagulation. There is no clear or sufficient information available to support discontinuation of anticoagulation once splanchnic vein thrombosis is stabilized; this is particularly applicable to those who also have MPNs. The potential choices for anticoagulation have been heparin (very short half-life), low-molecular-weight heparin (also short half-life), and warfarin (erratic INR levels outside the therapeutic range and need for frequent INR testing). There has been a shift toward using direct oral anticoagulants (DOACs) like apixaban and rivaroxaban [53]. With a DOAC, renal function must be monitored because dose reduction or discontinuation of the anticoagulant is required with significant chronic or acute kidney disease. Transjugular intrahepatic portosystemic shunt (TIPS) is a viable option after anticoagulation has been started. TIPS has become the standard of care for patients with splanchnic vein thrombosis with or without MPNs. Surgical portosystemic shunt placement has been mostly abandoned because of its high mortality rate. In MPN patients, unfortunately, there are high rates of TIPS thrombosis or stenosis and recurrent splanchnic vein thrombosis despite appropriate anticoagulation and TIPS placement. Most such patients will need re-intervention [54]. Despite medical therapy and interventional radiology, liver transplantation is necessary for approximately 10% of patients [54]. For patients with MPN, liver transplants could be more complicated and require interdisciplinary and patient consensus. For MPN patients with splanchnic vein thrombosis, in addition to the above-discussed interventions, cytoreductive treatment has been determined to show benefit in preventing rethrombosis [55].

**Cerebral venous sinus thrombosis (CVST).** CVST is a life-threatening form of thrombosis and a rare form of stroke. Patients with CVST most commonly present with headache, nausea, and vomiting, as well as papilledema, diplopia, and cognitive impairment. In the general population, major risk factors for CVST include the use of oral contraceptives, pregnancy, and thrombophilia. MPNs alone, in the absence of other known risk factors, have been associated with CVST [56,57], in which case CVST has worse clinical outcomes, a higher clot burden, and higher systemic thromboinflammatory markers than those who have CVST without an underlying MPN [57,58]. The acute treatment of CVST in the general population is prompt anticoagulation with unfractionated heparin or low-molecular-weight heparin. Even if there is a hemorrhagic transformation in the brain, heparin should be continued, because the hemorrhage is caused by the high pressures created by the occlusion of cerebral veins and sinuses. There are no evidence-based guidelines for the duration of anticoagulation, usually with a DOAC. In those with provoking factors, it would be reasonable to continue anticoagulation only until there is resolution of the thrombus by imaging and the provoking factor(s) have been eliminated. In CVST with MPNs, however, especially those with the *JAK V617F* mutation, patients can have a recurrence of thrombosis. Making sure there are no concurrent risk factors that can be reversed (e.g., oral contraceptive use, pregnancy, hormone-replacement therapy), continuing oral anticoagulation in these individuals for an indefinite period of time and giving them cytoreductive treatment to control blood counts would be a reasonable strategy.

## 9. Microvascular Disease in the Myeloproliferative Neoplasms

**Digital ischemia.** Digital ischemia can develop in MPN patients with unsuccessful or no treatment of arteriolar thrombosis [49]. Clinically, in these cases, the distal lower limb pulses are palpable, and ankle–brachial pressure indices are normal. Angiography can often show small arterial vessel occlusions in the arterioles of the distal lower limbs, while imaging of large- and medium-sized arteries may show only minimal or no significant atherosclerotic changes [59]. In some cases, patients can present with digital ischemia, cyanosis, or even early gangrene, and then undergo unsuccessful bypass surgery before the MPN is diagnosed.

Untreated or inappropriately treated erythromelalgia (see below), acrocyanosis, blue toe syndrome, or early digital gangrene can precede digital ischemia. However, fixed digital ischemia is also seen in the MPNs without antecedent symptoms and signs [12,60].

**Erythromelagia.** Two forms of this disorder have been described. Primary erythromelalgia is often caused by mutations in the *SCN9A*, *SCN10A*, and *SCN11A* genes, which encode for NaV1.7, NaV1.8, and NaV1.9, neuronal sodium channels. It is now considered to be a form of peripheral neuropathy, rather than a primarily vascular problem [61]. Secondary erythromelalgia occurs in patients with an underlying disease such as autoimmune disorders (systemic lupus erythematosus, multiple sclerosis) or, most prominently, a myeloproliferative neoplasm (especially ET and PV). In the primary form, erythromelalgia is more likely to present in both upper and lower limbs and is more likely to have a symmetrical distribution. In contrast, erythromelalgia in the MPNs typically presents with the predominant involvement of one foot, particularly on its plantar aspect. Given the fundamental differences between the two, including genetics, clinical presentation, and treatment, it has been suggested that the primary form should be named differently. Erythromelalgia in the MPNs usually develops acutely and is characterized by “burning” pain that can be severe, sometimes in patches on the sole of the foot with corresponding areas of erythema, warmth, and tenderness [62]. Oral treatments for the erythromelalgia of MPNs include aspirin (to which some patients respond well and quite promptly), selective serotonin reuptake inhibitors, anticonvulsants, calcium channel blockers, and tricyclic antidepressants (e.g., amitriptyline). If none of these drugs are effective, infusions of nitroprusside, lidocaine, or prostaglandins have been utilized [62]. In rare, completely refractory cases, sympathetic blocks, epidurals, and sympathectomy have been used with very variable responses.

**Cutaneous forms of microvascular disease.** Livedo racemosa and livedo reticularis are characterized by mottled, violaceous, fishnet-like, cyanotic areas surrounding a paler central core of the skin. These skin manifestations are caused by impaired blood flow to cutaneous microvessels of the area of skin involved [63]. The differences between livedo racemosa and reticularis are subtle [49]. Racemosa tends to be generalized (including the trunk, buttocks, and extremities), while reticularis is more localized to skin over the legs. The histologic characteristics of livedo racemose in ET have been shown to be platelet-mediated microvascular thrombosis, not vasculitis [64]. In contrast, reticularis is a physiologic phenomenon caused by reactive cutaneous vasoconstriction (e.g., in response to cold). Livedo racemosa is by no means diagnostic of MPN; several other disorders can cause it, including antiphospholipid syndrome, vasculitides, cholesterol embolism, cryoglobulinemia, cold agglutinin disease, and disseminated intravascular coagulation [63]. Raynaud syndrome has also been reported in ET.

## 10. Ocular Microvascular Disease in the Myeloproliferative Neoplasm

Ocular complications are common among patients with MPNs. The prevalence of ocular and neuro-ophthalmologic complications in the MPNs has been estimated to be between 7.5% and 25% in both treated and untreated patients [65]. Microvascular disturbances and hyperviscosity (in polycythemia vera) can promote retinal microvascular occlusions. A recent retinal imaging study of 40 patients with MPNs demonstrated changes in the microcirculation, with a reduced vascular density of the deep and superficial capillary plexuses. These were not found in healthy subjects. Whether or not retinal changes are predictive of large vessel thrombosis in patients with MPNs has yet to be determined [66].

## 11. Pathobiology of Thrombosis in the Myeloproliferative Neoplasms

The pathobiology of thrombosis in the MPNs is far more complex than just the erythrocytosis in PV, the thrombocytosis in ET, and the controversial question of leukocytosis contributing to thrombosis risk. Several recent publications have provided comprehensive reviews of the pathobiology of thrombosis in MPNs, involving interactions between a variety of factors, such as abnormally activated platelets, red cells, leukocytes, and vascular endothelial cells, the generation of microparticles, abnormal stimulation of non-myeloid inflammatory cells (T-lymphocytes, natural killer (NK) cells) that produce excessive inflammatory cytokines, overexpression of adhesive proteins, and even activation of the coagulation system [6,49,67,68,69,70]. Vascular endothelial cells are now recognized to be integral to the pathobiology of thrombosis in the MPNs. Endothelial cells, like hematopoietic cells, ultimately derive from mesodermal pluripotent stem cells. Not unexpectedly, therefore, endothelial cells in the splanchnic venous vasculature have been found to bear the JAK2 V617F mutation in JAK2-positive MPN individuals [6]. Neutrophil extracellular traps (NETs) are produced by inflammation, which, in turn, create an even more proinflammatory bone marrow microenvironment that favors the proliferation of mutant hematopoietic cells [6]. Overarching the abnormal activation and interactions of these cells and aberrant cytokine signaling are two major causes of thrombosis, namely aging and inflammation (i.e., thromboinflammation). The role of aging in thrombosis in the MPNs is likely to be the inflammatory response to aging.

## 12. Does Thrombosis Represent Progression of Disease in the Myeloproliferative Neoplasms?

Traditionally, the “progression of disease” in the MPNs has been defined as any of the following events: (1) transformation to myelofibrosis (MF), (2) transformation to acute leukemia, (3) transformation to myelodysplasia, (4) ET converting to PV, (5) ET converting to post-ET MF, and (6) PV converting to post-PV MF. Whether or not thrombosis is a harbinger of the progression of disease has been disputed. Supporting the addition of thrombosis as a contributor to the progression of the disease is the fact that it is the major cause of morbidity and mortality in the MPNs. To determine the impact of thrombosis on probabilities and trajectory of death and disease progression, a well-characterized international cohort of 1545 patients with PV was studied using a parametric Markov model. The results suggested thrombosis in PV to be a marker of aggressive disease biology or a disease-associated inflammatory state that is consequential to both thrombosis and disease progression [71].
